# The path to treating multiple-etiology dementia: Combining vascular risk control and disease-modifying neurodegenerative therapies

**DOI:** 10.1016/j.neurot.2026.e01068

**Published:** 2026-09-11

**Authors:** Adam de Havenon, Kevin N. Sheth, Carolyn A. Fredericks, Adam M. Brickman

**Affiliations:** 1 Department of Neurology, Yale University, New Haven, CT, USA; 2 Center for Brain and Mind Health, Yale University, New Haven, CT, USA; 3 Taub Institute for Research on Alzheimer's Disease and the Aging Brain, Department of Neurology, Columbia University, New York, NY, USA

**Keywords:** Dementia, Mixed-etiology dementia, Vascular dementia, Alzheimer's dementia

## Abstract

Multiple-etiology dementia (MED), typically characterized by coexisting vascular and neurodegenerative pathology, is the most common cause of late-life cognitive impairment. Autopsy and biomarker studies demonstrate frequent overlap between cerebral small vessel disease, stroke-related injury, amyloid-β deposition, and tau pathology, yet therapeutic development has largely proceeded within single pathway silos. Randomized trials support vascular risk reduction and multidomain lifestyle interventions as strategies that modestly improve cognitive trajectories. Separately, phase 3 trials of anti-amyloid monoclonal antibodies have established proof of principle that targeting amyloid-β can modestly slow clinical decline in early Alzheimer's disease. However, patients with mixed vascular and neurodegenerative disease are often excluded from these trials, limiting generalizability to real-world populations. We propose an integrated research roadmap for MED that prioritizes biomarker-enriched cohorts, combination vascular and neurodegenerative therapies, and pragmatic trials. A unified brain health framework that addresses both pathologic domains simultaneously offers the most plausible strategy to reduce the growing global burden of MED.

## Introduction

**Fig. 1:**
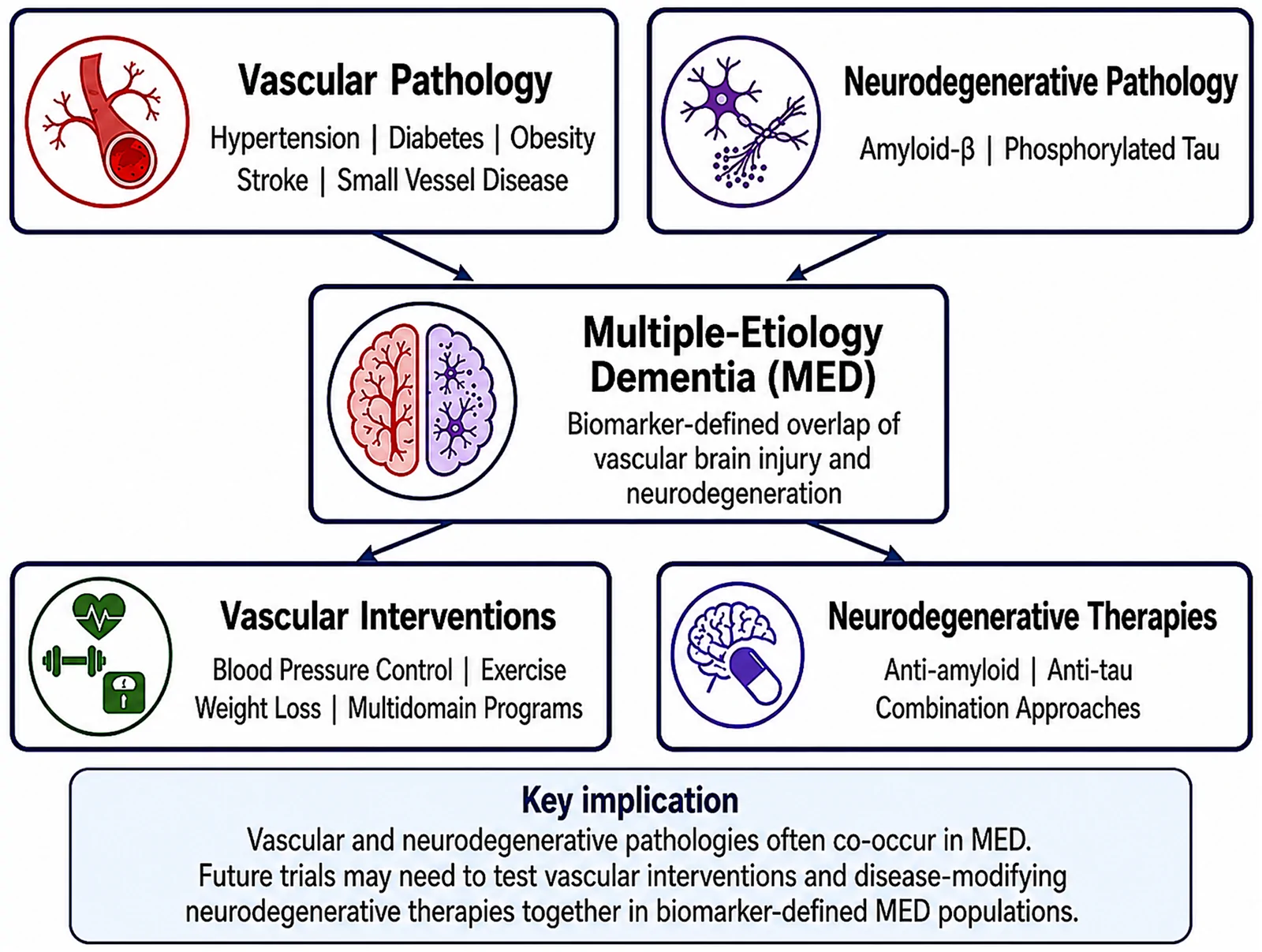
An integrated biological and therapeutic framework for multiple-etiology dementia.

The prevalence of Alzheimer's Disease-Related Dementias (ADRD) is anticipated to triple from roughly 50 million in 2025 to 150 million worldwide by 2050 [1]. Within the ADRD etiologies, multiple-etiology dementia (MED) represents a growing public health challenge [[2], [3], [4], [5]]. Although MED is also referred to as mixed dementia, mixed-etiology dementia, or multifactorial dementia, the National Institute of Neurological Disorders and Stroke currently uses the term “multiple-etiology dementia.” (6) MED is defined as a clinical dementia syndrome with multiple co-occurring neuropathologic processes, including combinations of cerebrovascular disease and neurodegenerative proteinopathies such as amyloid-β, phosphorylated tau, α-synuclein, TDP-43, or other pathologies [[6], [7], [8], [9]]. Thus, MED is not limited to Alzheimer's disease (AD) plus vascular disease, but may include AD with Lewy body disease, AD with limbic-predominant age-related TDP-43 encephalopathy (LATE), frontotemporal lobar degeneration with AD, or other combinations of pathologies [10] (see Fig. 1)

However, because the combination of AD and vascular pathology represents the overwhelming majority of MED, it will be the focus of this review article [[11], [12], [13], [14], [15], [16]]. In the United States, MED affects up to three million adults and is more prevalent in socioeconomically disadvantaged population [[17], [18], [19], [20], [21], [22]]. Individuals with MED also typically present with more severe cognitive impairment and experience faster progression compared to those with single-etiology dementia [11,[23], [24], [25]].

We discuss therapeutic options for the prevention or treatment of MED, their interplay with both Alzheimer's and vascular pathology, and a roadmap for future MED therapeutic research. We will not cover diagnosis, epidemiology, or mechanisms of MED in depth, as these have been reviewed elsewhere [[6], [7], [8], [9]]. No randomized clinical trials (RCTs) have been designed specifically to target MED; therefore, we extrapolate from trials focused on either neurodegenerative or vascular pathology. We focus on modifiable midlife and late-life vascular risk factors for which RCTs demonstrate impact on cognitive outcomes or dementia risk. Early-life determinants, hearing or visual loss, structural or societal exposures, and injury prevention are beyond the scope of this review [4].

## Vascular-targeted interventions

Poor control of vascular risk factors, principally hypertension, diabetes, physical inactivity, and obesity, is strongly linked to the risk of MED in observational studies [[26], [27], [28], [29], [30], [31]]. Because vascular risk factors are highly prevalent, particularly in mid and later life, they have been the subject of multiple RCTs.

### Hypertension control

The Systolic Blood Pressure Intervention Trial–Memory and Cognition in Decreased Hypertension (SPRINT-MIND) provides the strongest randomized evidence that intensive blood pressure control influences cognitive outcomes [32]. In this 2019 ancillary study to the SPRINT trial (which had a cardiovascular outcome), 9361 adults aged ≥50 years at elevated cardiovascular risk but without diabetes or prior stroke were randomized to intensive systolic blood pressure lowering (<120 mmHg) versus standard treatment (<140 mmHg) [33]. Over a median follow-up of 5.1 years, intensive treatment reduced the incidence of mild cognitive impairment (MCI) by approximately 19% (hazard ratio [HR] 0.81; 95% CI 0.69–0.95). The combined outcome of MCI or probable dementia was reduced by about 15% (HR 0.85; 95% CI 0.74–0.97). The reduction in probable dementia alone had a similar favorable effect but did not reach statistical significance (HR 0.83; 95% CI 0.67–1.04).

More recently, a large cluster-randomized trial conducted in rural China (the China Rural Hypertension Control Project [CRHCP]) extended these findings in a pragmatic, community-based setting [34]. In this 2025 study of 33,995 adults with hypertension, villages were randomized to an intensive blood pressure management program targeting systolic blood pressure <130 mmHg versus usual care. Over 4.0 years of follow-up, intensive blood pressure control lowered the risk of incident all-cause dementia by 15% (HR 0.85; 95% CI 0.75–0.97), achieved with a significant 22 mmHg reduction in systolic blood pressure compared with usual care.

Together, the SPRINT-MIND and CRHCP trials provide complementary, and very consistent, evidence that blood pressure lowering reduces the incidence of cognitive impairment and dementia, supporting its central role in prevention strategies for MED. The CRHCP trial also suggests that beyond pharmacologic efficacy, the critical next step is to identify and scale effective implementation strategies at the individual, health system, and population levels to achieve sustained blood pressure control and maximize cognitive benefit.

### Glycemic control

RCTs examining the effect of intensive glycemic control in diabetics on cognitive outcomes have been less definitive than the blood pressure trials. In the Action to Control Cardiovascular Risk in Diabetes–Memory in Diabetes (ACCORD-MIND) study [35], a 2011 substudy of the larger Action to Control Cardiovascular Risk in Diabetes (ACCORD) trial [36], 2977 participants with longstanding type 2 diabetes (mean age 62 years; median diabetes duration approximately 10 years) were randomized to intensive glycemic control (target glycated hemoglobin [HbA1c] <6.0%) versus standard therapy (target 7.0–7.9%). After a median 5 years of follow-up, there was no significant difference in cognitive performance on the Digit Symbol Substitution Test, the primary cognitive endpoint. Similarly, in the Action in Diabetes and Vascular Disease: Preterax and Diamicron Modified Release Controlled Evaluation (ADVANCE) trial [37], 11,140 participants with type 2 diabetes were randomized to intensive glucose control targeting HbA1c ≤ 6.5% versus standard care. Intensive therapy did not demonstrate a clear reduction in dementia incidence or cognitive decline.

Despite preliminary data suggesting that the glucagonlike peptide-1 (GLP-1) receptor agonist and sodium-glucose cotransporter-2 inhibitors (SGLT2) antidiabetic medications may reduce dementia risk [38,39], the phase 3 EVOKE and EVOKE + trials of oral semaglutide in early symptomatic Alzheimer's disease did not demonstrate a significant reduction in clinical disease progression on the Clinical Dementia Rating-Sum of Boxes (CDR-SB) score despite favorable effects on several biomarkers [40].

Together, these trials suggest that while diabetes is an established risk factor for cognitive decline, intensive glycemic control alone does not reduce incident dementia meaningfully. One explanation is that most participants had longstanding type 2 diabetes, when cerebrovascular and neurodegenerative injury may already have become established and less amenable to modification. These findings also suggest that hyperglycemia is only one component of vascular risk, and that meaningful reductions in dementia risk will likely require simultaneous management of multiple vascular factors, including blood pressure, obesity, physical inactivity, and dyslipidemia, as reflected in successful multidomain intervention trials.

### Physical activity

Several RCTs evaluated physical activity interventions and cognitive outcomes, but have been modest in size. Lautenschlager et al. randomized 170 older adults with subjective memory impairment to a 24-week home-based physical activity program versus usual care and observed a small but statistically significant improvement in Alzheimer's Disease Assessment Scale-Cognitive Subscale (ADAS-Cog) at 18 months (mean between-group difference 0.26 points) [41]. In patients with clinically diagnosed mild Alzheimer's disease (AD), Hoffmann et al. randomized 200 individuals with mild AD to 16 weeks of supervised moderate-to-high–intensity aerobic exercise versus usual care. The primary cognitive endpoints were neutral, but high-adherence participants showed signal for benefit [42].

Similarly, the SYNERGIC trial randomized 175 older adults with mild cognitive impairment to aerobic-resistance exercise, cognitive training, both interventions, or control [43]. Although the smaller sample size does not provide conclusive results, SYNERGIC found that the combination of exercise and cognitive training produced the greatest improvement in global cognition over 20 weeks, supporting the concept that multidomain interventions may provide greater cognitive benefit than single-component strategies. More recently, the 2025 EXERT trial randomized 296 sedentary older adults with amnestic mild cognitive impairment to moderate-intensity aerobic training versus stretching for 12 months. The primary cognitive outcome (ADAS-Cog-Exec) did not differ between groups. Collectively, these RCTs demonstrate small effect sizes but are limited by relatively small samples, short duration, and cognitive test endpoints rather than adequately powered dementia-prevention designs.

### Weight loss

RCTs specifically testing obesity prevention or intentional weight loss for cognitive or dementia outcomes are limited. Small to moderate-sized RCTs (often n ˜ 50–100) in obese older adults, including those with mild cognitive impairment, suggest that intentional weight loss through dietary and behavioral interventions may modestly improve memory and executive function, though these studies are generally underpowered and heterogeneous in design [[44], [45], [46]]. The largest relevant trial, Look AHEAD [46], tested a long-term intensive lifestyle weight-loss intervention in 978 obese or overweight diabetics but did not demonstrate a clear cognitive benefit. While obesity is associated with dementia risk in observational studies, randomized evidence that weight loss or obesity prevention reduces incident cognitive decline or dementia remains limited and inconclusive.

### Multidomain vascular interventions

The strongest efficacy signal for a multidomain intervention comes from the 2015 Finnish Geriatric Intervention Study to Prevent Cognitive Impairment and Disability (FINGER) RCT, which randomized 1260 at-risk older adults [[47], [48], [49], [50], [51], [52], [53], [54], [55], [56], [57], [58], [59], [60], [61], [62], [63], [64]] to a 2-year program combining diet counseling, exercise training, cognitive training, and vascular risk-factor monitoring versus general health advice [65]. In the modified intention-to-treat analysis (94.5% of participants with post-baseline data), global cognition measured by a Neuropsychological Test Battery total z-score improved in both groups, but the intervention arm improved more (mean change at 2 years was 0.20 (SE 0.02) vs 0.16 (SE 0.01), corresponding to a group difference of 0.022 z-score units per year, 95% CI 0.002–0.042, p = 0.030). Domain-level effects were most evident for executive function and processing speed, which are the domains most implicated in vascular cognitive impairment [[66], [67], [68], [69]].

The 2025 US. Study to Protect Brain Health Through Lifestyle Intervention to Reduce Risk (US POINTER) RCT was designed to test whether FINGER-like benefits generalize to a larger, more diverse U.S. cohort [70]. It enrolled and randomized 2111 cognitively unimpaired but at-risk adults [[47], [48], [49], [50], [51], [52], [53], [54], [55], [56], [57], [58], [59], [60], [61], [62], [63], [64],^71^,^72^] to a Structured (STR) vs Self-Guided (SG) multidomain program; both arms targeted exercise, nutrition, cognitive/social challenge, and health monitoring, but STR added substantially more support/accountability. At 2 years, both groups improved, but the STR group improved more on the primary global cognitive composite: +0.029 SD per year vs SG (95% CI 0.008–0.050; p = 0.008); executive function also favored STR (+0.037 SD per year; 95% CI 0.010–0.064), while memory did not differ between groups.

In the small FINGER PiB-PET substudy (n = 47), 42% of participants were visually amyloid-positive, which was associated with worse executive function, but not with a higher Fazekas score or differences in vascular risk factors. This finding further supports the concept that their intervention may benefit MED [73]. In U.S. POINTER, an imaging ancillary study similarly demonstrated biological heterogeneity in 1502 participants, of which 29% had positive amyloid-β status on PET [74]. Aβ+ participants had higher white matter hyperintensity volume, consistent with the findings in the FINGER substudy.

Although the magnitude of benefit in both RCTs was modest and the primary outcome was change in cognitive performance rather than incident dementia, the findings were robust and derived from large, well-conducted trials. Neither FINGER nor US POINTER was designed to isolate the independent effects of individual components, limiting causal inference regarding which domains drive benefit. Nonetheless, the results demonstrate that a scalable, multidomain lifestyle intervention can produce measurable improvements in cognitive trajectories over two years and that greater intervention intensity and support are associated with larger effects.

### Untested vascular interventions

Because cognitive impairment and dementia affect up to a third of stroke survivors [[47], [48], [49],[75], [76], [77]], the identification and treatment of stroke risk factors such as atrial fibrillation or hyperlipidemia could theoretically reduce MED [50]. Similarly, preventing traumatic brain injury (TBI), which is a form of brain and vascular injury, through more widespread use of helmets or seat belts could also theoretically reduce MED [51,52]. However, at a population level, stroke and TBI are not prevalent enough to be studied in clinical trials as a treatment to reduce the risk of dementia or MED [53,54].

## Neurodegenerative interventions

Recent phase 3 RCTs of anti-amyloid monoclonal antibodies have demonstrated that disease-modifying therapy for early AD is biologically and clinically achievable. In the Clarity AD trial, lecanemab was tested in 1795 participants with early AD and slowed decline on the Clinical Dementia Rating-Sum of Boxes (CDR-SB) by 0.45 points over 18 months compared with placebo (−1.21 vs −1.66; 27% relative slowing; p < 0.001) [55]. Similarly, in the TRAILBLAZER-ALZ 2 trial, donanemab was evaluated in 1736 participants with early symptomatic AD and intermediate tau burden and reduced decline on the integrated Alzheimer's Disease Rating Scale (ADRS) by approximately 35% relative to placebo at 18 months, with parallel effects on CDR-SB [56]. Both agents reduced amyloid burden on positron emission tomography but were associated with amyloid-related imaging abnormalities (ARIA) adverse events, including edema and microhemorrhage [57].

Overall, these trials establish proof of principle that targeting amyloid can alter AD trajectories. Ongoing and planned studies are extending this approach to earlier preclinical populations, combination strategies, and tau-targeting therapies, including multiple phase 2 and phase 3 anti-tau monoclonal antibody trials and next-generation amyloid-directed agents [58], reflecting continued efforts to refine and amplify disease-modifying effects in AD-related neurodegeneration, which will be highly applicable to MED populations.

However, the recent anti-amyloid trials largely excluded patients with established vascular disease, including prior stroke, cerebral amyloid angiopathy, or severe cerebral small vessel disease such as white matter hyperintensities (WMH) or microhemorrhages [55,56,[59], [60], [61], [62]]. In a Mayo Clinic Study of Aging cohort, which included participants with mild cognitive impairment or mild dementia and an amyloid-positive PET, only 8.0% were eligible to receive lecanemab based on clinical trial criteria [63]. Although most individuals had multiple exclusionary criteria, the neuroimaging criteria of prior stroke or severe WMH were among the most common [63,64,71,72]. Further, the Appropriate Use Guidelines for anti-amyloid therapy also exclude subjects with prior stroke or severe WMH [[78], [79], [80], [81]]. If MED patients with prior stroke or severe chronic microvascular disease continue to be excluded from trials of anti-amyloid or tau therapies, we will not understand the risk of these therapies for this population, and they will not be able to derive potential benefit for the neurodegenerative component of their pathology.

## Synthesis of existing evidence

RCT data relevant to MED support a multidomain therapeutic framework but remain uneven across mechanisms (Table 1). Intensive blood pressure control provides the most consistent vascular interventional signal, whereas intensive glycemic control has not reduced dementia incidence despite clear vascular benefits. Lifestyle and multidomain interventions yield modest but reproducible cognitive advantages, particularly in executive function, with favorable safety and scalability profiles. In contrast, anti-amyloid monoclonal antibodies slow clinical progression in early AD but carry higher treatment burden and risk.

**Table 1:** Randomized trial evidence relevant to multiple-etiology dementia.

Intervention	Target	Trials	Scope	Effect size	Risk profile	Feasibility
Intensive blood pressure control	Vascular	SPRINT-MIND; CRHC	Middle-aged and older adults at vascular risk; hypertensive populations	15–19% relative reduction in MCI or dementia (HR ∼0.81–0.85); directionally favorable dementia signal	Low–moderate (hypotension, syncope, electrolyte abnormalities)	High; widely implementable in primary care and community settings
Intensive glycemic control	Vascular (metabolic)	ACCORD-MIND; ADVANCE	Older adults with longstanding type 2 diabetes	No significant reduction in cognitive decline or dementia	Moderate (hypoglycemia, weight gain, mortality signal in ACCORD)	Moderate; implementable but limited cognitive efficacy evidence
Physical activity	Vascular + Neurodegenerative (pleiotropic)	EXERT; SYNERGIC; small RCTs	Older adults with subjective impairment, MCI, or mild AD	Small cognitive effects; neutral primary outcomes in larger trials; signal in high adherence groups	Low	High; scalable but adherence-dependent
Weight loss	Vascular (metabolic)	Look AHEAD; small behavioral RCTs	Obese/overweight older adults, often with diabetes or MCI	Modest cognitive improvements in small trials; no clear dementia reduction in large RCT	Low–moderate	Moderate; behavioral intensity required
Multidomain lifestyle intervention	Vascular; multifactorial	FINGER; US POINTER	At-risk older adults [[47], [48], [49], [50], [51], [52], [53], [54], [55], [56], [57], [58], [59], [60], [61], [62], [63], [64],^71^,^72^], cognitively normal or mildly impaired	Small but statistically robust cognitive benefit (∼0.02–0.03 SD/year advantage); executive function most responsive	Low	Moderate–high; scalable with structured support systems
Anti-amyloid monoclonal antibodies (lecanemab, donanemab)	Neuro-degenerative (amyloid)	Clarity AD; TRAILBLAZER-ALZ 2	Early symptomatic AD with biomarker confirmation	∼27–35% relative slowing of clinical decline over 18 months; CDR-SB difference ∼0.45 points (lecanemab)	Moderate–high (ARIA-E/H, infusion reactions)	Limited; requires biomarker confirmation, infusion infrastructure, MRI surveillance
Anti-tau/Next-Generation disease-modifying therapies	Neuro-degenerative (tau pathology)	Ongoing phase 2–3 trials	Early AD and biomarker-enriched populations	Emerging; proof-of-concept stage	Unknown to moderate (agent-specific)	Currently limited; research setting
Combination therapy (Proposed future strategy)	Integrated vascular + neuro-degenerative	Not yet tested in MED	Biomarker-defined MED populations	Hypothesized additive/synergistic effects; not yet quantified	Dependent on components	Moderate; requires coordinated trial infrastructure

## Challenges in MED research and treatment

### Structural fragmentation in MED care and research

The most difficult challenge for MED treatment is that the cognitive and vascular clinical and research spheres are traditionally siloed [[82], [83], [84]]. Within United States neurology subspecialties, there is little overlap in the training requirements for Vascular Neurology and Behavioral/Cognitive Neurology [[85], [86], [87]]. Consequently, patients with MED often require expertise spanning vascular risk factor management, stroke prevention, neurodegenerative diagnosis, biomarker interpretation, and disease-modifying therapies, yet these domains are frequently delivered in separate clinical settings. Similarly, the clinical care of patients with dementia is distributed across neurology, primary care, and psychiatry, each with differing expertise and priorities [[88], [89], [90], [91]]. These structural divisions underscore the need for greater cross-disciplinary training and collaborative care models that integrate vascular neurologists, behavioral/cognitive neurologists, primary care physicians, and other specialists to provide comprehensive brain health care for patients with MED.

In the clinical research sphere, the recent major anti-amyloid randomized trials were not designed to systematically assess or modify vascular risk factors at follow-up visits [55,56,[59], [60], [61], [62]]. Similarly, randomized trials of vascular interventions to prevent dementia have not measured or engaged with neurodegenerative pathology such as amyloid-β or phosphorylated tau [32,65,70,92]. The result is that patients with MED are often studied or treated within single-disease paradigms that incompletely address the spectrum of underlying pathology.

A holistic concept of “brain health” has recently emerged as a strategic priority for both the World Health Organization, American Heart Association, and American Academy of Neurology [[93], [94], [95], [96], [97]]. This framework is directly relevant to MED because it recognizes that cognitive decline and dementia often reflect the cumulative effects of both vascular and neurodegenerative injury rather than a single disease process. The brain health model also shifts the focus from treating established dementia to preserving cognitive function across the lifespan. Rather than targeting a single pathology after symptoms develop, a brain health approach supports earlier, multidomain interventions which would be particularly effective for MED.

## Future MED research roadmap

### Biomarker-defined trial populations

Future research in MED must move beyond single-pathology paradigms and toward integrated, mechanism-informed trial designs. First, RCTs should be explicitly enriched for participants with both vascular and neurodegenerative biomarkers. Biomarker-defined MED cohorts, using amyloid and tau imaging or blood biomarkers and magnetic resonance imaging markers of vascular injury such as prior stroke or small vessel disease, could simultaneously target vascular and neurodegenerative pathways. Such enrichment may also improve statistical efficiency by increasing the likelihood that participants harbor both treatable vascular and neurodegenerative pathology.

### Combination therapeutic strategies

Combination therapy trials represent a particularly high-priority direction, pairing aggressive vascular risk factor modification (e.g., intensive blood pressure lowering, lipid optimization, structured exercise) with disease-modifying neurodegenerative agents such as anti-amyloid or anti-tau monoclonal antibodies. The greatest opportunity for combination therapies may lie in biomarker-defined individuals with subjective cognitive impairment or mild cognitive impairment who demonstrate evidence of both vascular injury and neurodegenerative pathology, before irreversible brain injury has accumulated. Such designs would permit evaluation of additive or synergistic therapeutic effects while determining whether vascular risk reduction modifies the efficacy or safety of disease-modifying neurodegenerative therapies. Emerging data suggest that hypertension control may be one of the most important modifiable risk factors for ARIA [98,99].

### Trial design and implementation

Future MED research must incorporate early intervention windows and longer follow-up horizons. Trials should also prioritize the clinical outcomes of incident mild cognitive impairment or dementia. When clinical outcomes such as incident MCI or dementia are not feasible, validated imaging biomarkers and disease progression metrics should be prioritized over short-term neuropsychological change alone. Implementation science will also be critical, as scalable multidomain interventions must be deliverable in socioeconomically diverse populations where MED burden is greatest.

MED trials should also account for the heterogeneity of baseline vascular risk management by carefully characterizing existing pharmacologic therapies, lifestyle behaviors, and vascular risk factor control, and incorporating these variables into trial design and prespecified subgroup analyses. They should build upon contemporary guideline-directed vascular care rather than replace it, evaluating optimized treatment targets, implementation strategies that improve achievement of vascular risk factor control, and the addition of disease-modifying neurodegenerative therapies to standard vascular management. They could benefit from statistical designs capable of evaluating combination effects without becoming prohibitively large. Full factorial designs can estimate the independent and interactive effects of intervention components, as illustrated by multidomain trials such as SYNERGIC [43]. Such approaches may help distinguish additive from synergistic effects while maintaining feasibility, interpretability, and alignment with regulatory expectations.

### Collaborative infrastructure

Finally, advancing MED will require new collaborative infrastructure that bridges vascular neurology, behavioral neurology, geriatrics, primary care, neuroradiology, and clinical trial networks. Rather than conducting separate vascular and neurodegenerative trials, future platforms should be designed to evaluate multidomain interventions within shared biomarker-defined populations. Such an approach would improve efficiency, enhance generalizability, and better reflect the biology of MED.

## Conclusion

MED represents a common and consequential form of ADRD in which vascular and neurodegenerative pathologies converge to accelerate cognitive decline. Advancing the field will require biomarker-enriched trials that combine aggressive vascular risk reduction with disease-modifying neurodegenerative therapies. A strategy that bridges traditional silos offers the most coherent and scientifically grounded pathway to mitigate the expanding global burden of MED.

## Author contributions

All authors contributed equally to the manuscript.

## Disclosures

Dr. De Havenon has received consultant fees from Integra and Novo Nordisk, royalty fees from UpToDate, and has equity in TitinKM and Certus. Dr. Sheth reports compensation from; compensation from Sense and Zoll, for data and safety monitoring services; compensation from Cerevasc for consultant services; compensation from Rhaeos for consultant services; compensation from Certus for consultant services; a patent pending for Stroke wearables licensed to Alva Health. Dr. Brickman is an advisor/consultant for CogniMark, Tau Biosciences, Cognito Therapeutics, IQVIA, CogState, Cognition Therapeutics), he has received speaker fees from Novo Nordisk and Celdara, and has equity in CogniMark and Tau Biosciences. He holds a patent for white matter hyperintensity quantification (US Patent US9867566B2) and has a patent pending for cerebral microbleed detection (U.S. provisional patent application no.: 63/146,958).

## Sources of Funding

Dr. De Havenon reports NIH/NINDS funding (UG3NS130228, R01NS, R21NS138995). Dr. Sheth by NIH-NINDS (U01NS106513, R01NS11072, R01NR018335, R01EB301114, R01MD016178, R03NS112859, U24NS107215, U24NS107136), and American Heart Association (17CSA33550004). Dr. Brickman reports funding from NIH (UG3AG090558, P30AG066462, R01AG054070, R01AG072474, R01AG076383, RF1AG079519), Department of Defense (TP230352), and Alzheimer's Association (22-AAIIA-970107, VCID-UMD-26-1530947).
